# Electrochemical Decalcification–Exfoliation of Two-Dimensional Siligene, Si_*x*_Ge_*y*_: Material Characterization and Perspectives for Lithium-Ion Storage

**DOI:** 10.1021/acsnano.3c00658

**Published:** 2023-06-07

**Authors:** Evgeniya Kovalska, Bing Wu, Liping Liao, Vlastimil Mazanek, Jan Luxa, Ivo Marek, Luc Lajaunie, Zdenek Sofer

**Affiliations:** † Department of Inorganic Chemistry, University of Chemistry and Technology Prague, Technická 5, 166 28 Prague 6, Czech Republic; ‡ Department of Engineering, Faculty of Environment, Science and Economy, University of Exeter, Exeter, EX4 4QF, United Kingdom; § Departamento de Ciencia de los Materiales e Ingeniería Metalúrgica y Química Inorgánica, Facultad de Ciencias, Universidad de Cádiz, Campus Río San Pedro S/N, Puerto Real, Cádiz, 11510 Spain; ∥ Instituto Universitario de Investigación de Microscopía Electrónica y Materiales (IMEYMAT), Universidad de Cádiz, Campus Río San Pedro S/N, Puerto Real, Cádiz, 11510 Spain

**Keywords:** silicene, germanene, low-hydrogenated siligene, top-down synthesis, electrochemical exfoliation, lithium-ion storage, lithium-ion battery

## Abstract

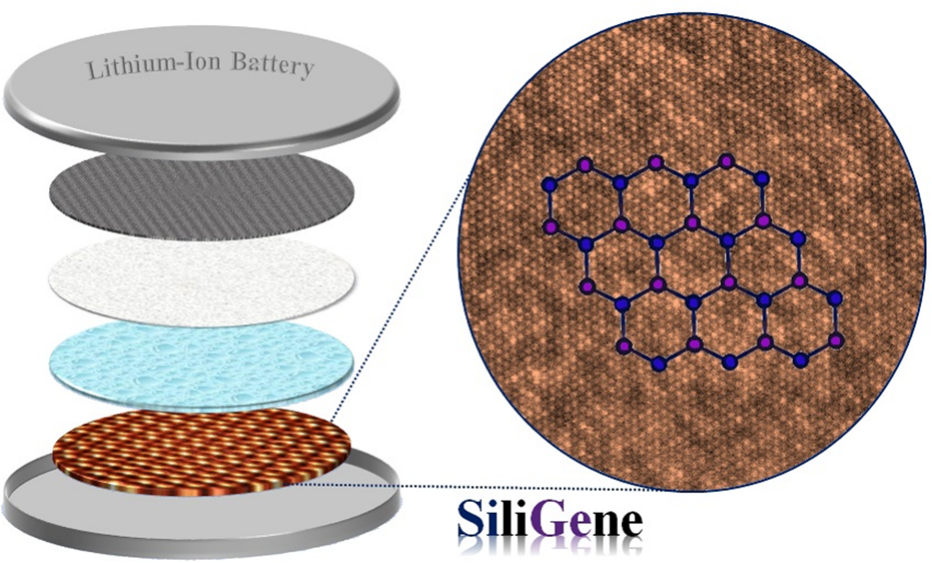

A two-dimensional (2D) silicene–germanene alloy, siligene (Si_*x*_Ge_*y*_), a single-phase material, has attracted increased attention due to its two-elemental low-buckled composition and unique physics and chemistry. This 2D material has the potential to address the challenges caused by low electrical conductivity and the environmental instability of corresponding monolayers. Yet, the siligene structure was studied in theory, demonstrating the material’s great electrochemical potential for energy storage applications. The synthesis of free-standing siligene remains challenging and therefore hinders the research and its application. Herein we demonstrate nonaqueous electrochemical exfoliation of a few-layer siligene from a Ca_1.0_Si_1.0_Ge_1.0_ Zintl phase precursor. The procedure was conducted in an oxygen-free environment applying a −3.8 V potential. The obtained siligene exhibits a high quality, high uniformity, and excellent crystallinity; the individual flake is within the micrometer lateral size. The 2D Si_*x*_Ge_*y*_ was further explored as an anode material for lithium-ion storage. Two types of anode have been fabricated and integrated into lithium-ion battery cells, namely, (1) siligene–graphene oxide sponges and (2) siligene–multiwalled carbon nanotubes. The as-fabricated batteries both with/without siligene exhibit similar behavior; however there is an increase in the electrochemical characteristics of SiGe-integrated batteries by 10%. The corresponding batteries exhibit a 1145.0 mAh·g^–1^ specific capacity at 0.1 A·g^–1^. The SiGe-integrated batteries demonstrate a very low polarization, confirmed by their good stability after 50 working cycles and a decrease in the solid electrolyte interphase level that occurs after the first discharge/charge cycle. We anticipate the growing potential of emerging two-component 2D materials and their great promise for energy storage and beyond.

Emerging two-dimensional (2D) materials with their remarkable properties have driven development for advanced energy storage systems. The superiority of 2D materials lies in their geometry and dimension that drastically differ in the materials’ properties from the corresponding layered bulk counterparts. Recently, 2D materials, a family of more than 150 members (e.g., graphene, pnictogens, MXenes, transition metal dichalcogenides, 2D polymers), have been replenished with a van der Waals structure composed of two elements, namely, silicon (Si) and germanium (Ge).^1^ This 2D derivative type of single-phase material promises to address the complexities caused by low electrical conductivity and environmental instability of monoelemental 2D layers toward the exploitation of high-performance energy storage.

For several decades, the SiGe structures have been known in their alloy form, crystallizing in a rather diamond-like lattice,^2^ which demonstrates random atom distributions.^3^ The determination of bulk lattice parameters across the so-called Si_1–*x*_Ge_*x*_ system has been previously studied, focusing on the variation of lattice parameters (bond lengths and bond angles) under different temperature conditions.^4^ This can influence the bond angle change and thus lead to different atom distributions within the lattice structure. Understanding the lattice composition has led to the implementation of SiGe in modern microelectronics as a hetero-bipolar transistor replacing pure Si versions. In addition, contrary to the spatial confinement of charge carrier systems like a plane Si/SiO_2_ configuration, the SiGe structure possesses a lower effective mass of the electrons, and band alignment can be tuned via bandgap engineering. Also the energy band is easily split into elastically strained layers.

The aforementioned advantages have been employed to exploit the 2D SiGe material. Several theoretical studies have reported a stable 2D hexagonal SiGe lattice with a low buckled structure based on density functional theory (DFT), analyzing the stability and nonlinear elasticity of the material.^5^ The in-plane hybrid called siligene and its hydrogenated forms were predicted by DFT.^6^ The authors studied materials structure and electronic properties, demonstrating a chair configuration as an energetically favorable form for monolayers with a bandgap of ∼0.6 eV. The other first-principles study has shown 2D SiGe as a promising anode material for sodium- and potassium-ion storage.^7^ The authors confirmed the thermal and dynamic stabilities of the 2D SiGe anodes that can provide stable voltage profiles and high theoretical capacities: 532 mAh·g^–1^ for Li^+^ or K^+^ and 1064 mAh·g^–1^ for Na^+^ storage. As for lithiation, for instance, Li^+^ intercalation between the interlayers of 2D SiGe enables the accommodation of 2 lithium ions per layer, with additional alloying reactions allowing for a theoretical capacity of 2920 mAh g^–1^. The experimental study on successful liquid exfoliation of the 2D SiGe hybrid was reported in 2021.^1^ In this work, researchers prepared samples with different ratios of Si and Ge components and showed their performance as an anode in lithium-ion batteries (LIBs). It has to be noted that these materials were demonstrated as 2D, but the corresponding scanning electron microscopy (SEM) analyses showed flakes with a thickness of dozen of micrometers and more, implicating rather the bulk layered SiGe structure after decalcification than a SiGe (mono)few-layer. Therefore, as-discussed LIBs cannot be categorized as 2D materials-based batteries, and thus their good electrochemical performance is due to the bulk originality of layered SiGe. Moreover, to the best of our knowledge, syntheses of siligene by the electrochemical exfoliation method, neither monolayer nor a few-layer, have not been reported yet.

Herein, we demonstrate a successful synthesis of few-layer 2D Si_*x*_Ge_*y*_ nanosheets with low-level hydrogenation (namely, SiGe or siligene) by controlled electrochemical exfoliation of a Zintl phase Ca_1.0_Si_1.0_Ge_1.0_ crystal in a nonaqueous environment. The procedure was carried out in the electrolyte composed of 0.03 M tetrabutylammonium perchlorate (TBAClO_4_) in acetonitrile employing a −3.8 V potential. A comprehensive morphological and structural analysis of obtained siligene was performed by a complex of microscopic and spectroscopic techniques such as high-resolution transmission electron microscopy assisted with selected area electron diffraction (HR-TEM-SAED), high-resolution scanning transmission electron microscopy assisted with high-angle annular dark-field (HR-STEM-HAADF), and coupled with energy-dispersive X-ray spectroscopy (EDS) analysis, X-ray diffraction (XRD), and X-ray photoelectron spectroscopy (XPS). In addition, the optical features of the exfoliated materials were studied using Raman, ultraviolet–visible (UV–vis) and photoluminescence (PL) spectroscopies. The siligene nanosheets were used for the fabrication of lithium storage electrode material in two combinations with reduced graphene oxide sponge (rGOS) and multiwalled carbon nanotubes (MWCNTs). Both electrodes were used as anode material for LIBs. Their electrochemical performance was tested by cyclic voltammetry (CV) and electrochemical impedance spectroscopy (EIS) and compared with pure rGOS- and MWCNTs-based LIBs. This research demonstrates a two-component single-phase 2D structure, providing an idea for the development of efficient energy storage materials.

## Results and Discussion

### Synthesis and Characterization of the Materials

**Figure 1:**
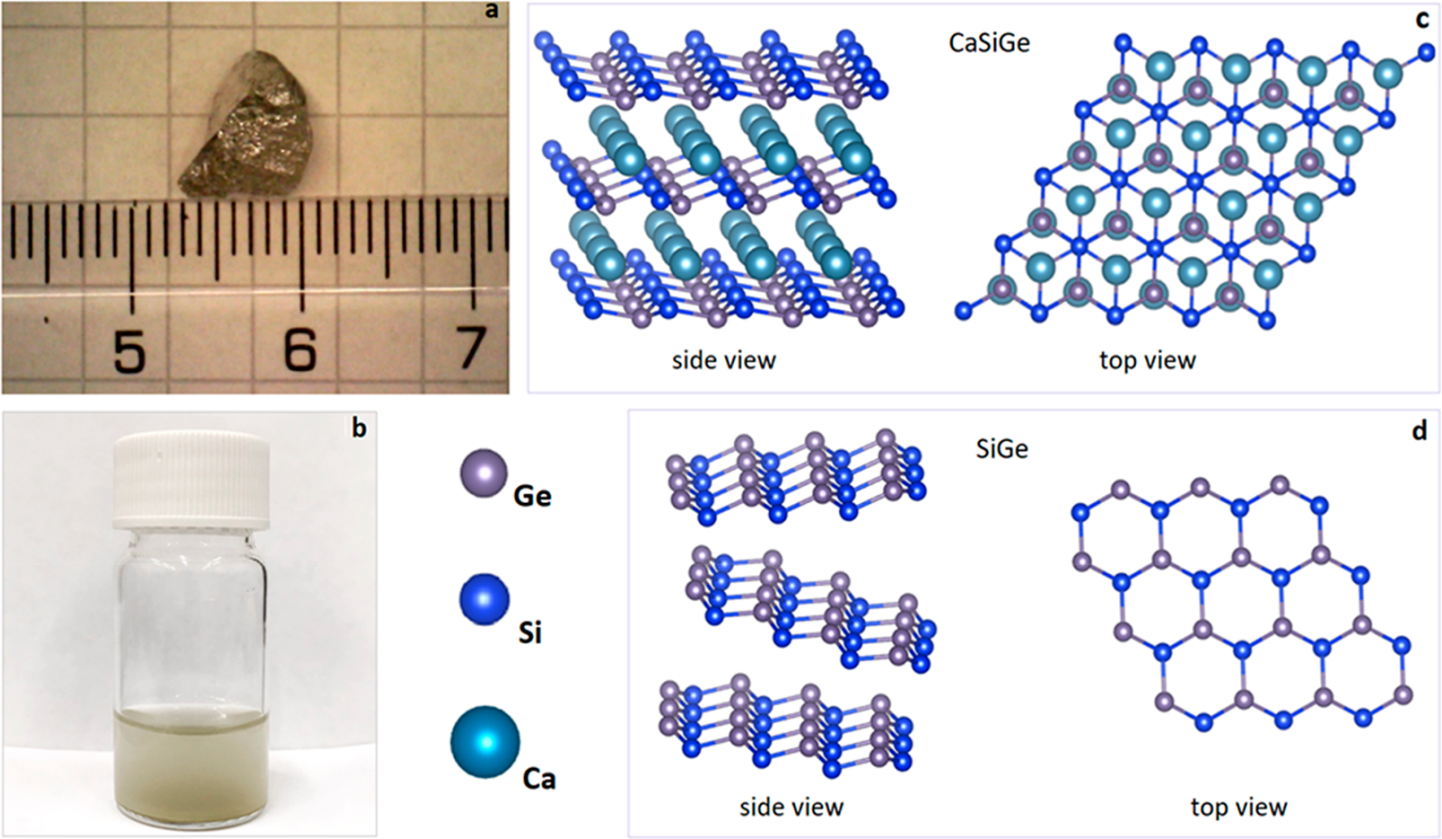
Digital photographs and schematic illustrations of the CaSiGe crystal (a, c) and its exfoliated form, SiGe–siligene, in acetonitrile (b, d). Schematics demonstrate the side and top views of the crystal and siligene structure.

Synthesis of a few-layer siligene was carried out by cathodic electrochemical exfoliation of Zintl phase material CaSiGe (Figure 1a) in a nonaqueous electrolyte (0.03 M TBAClO_4_ in acetonitrile). The oxygen-free environment was maintained by continuously purging argon directly into the electrolyte. The process of exfoliating 2D SiGe is founded on the decalcification–intercalation mechanism, whereby Ca undergoes reduction–oxidation reactions. The procedure has been separated into three stages applying different voltages, namely, −2.0, −2.87, and −3.8 V. Figure S1a demonstrates the first two stages of this mechanism, which involve (I) the accumulation of tetrabutylammonium cations (TBA^+^) at −2.0 V toward the layered CaSiGe and (II) decalcification–intercalation induced by two simultaneous processes: reduction of Ca^2+^ and TBA^+^ propagation at −2.87 V. This voltage potential is responsible for the reduction of Ca^2+^ cations, leading to their interaction with ClO^4–^ anions in the electrolyte medium, resulting in the formation of Ca(ClO_4_)_2_. Subsequent SiGe exfoliation (stage III) was the longest 5 h stage carried out at −3.8 V. This potential was sufficient to initiate the decalcification–intercalation processes and break the bonds in CaSiGe. The supplementary plot depicted in Figure S1b illustrates the temporal evolution of the reaction over the course of 1 h of exfoliation, as displayed by the current as a function of time. The electrochemical exfoliation process resulted in a nearly 100% yield of siligene sheets dependent on the initial quantity of CaSiGe crystal available. The exfoliated material in the electrolyte was transferred to a vial for a further 30 min of ultrasonication. This was followed by two-step washing via vacuum filtration: first, using 0.01 N acetic acid, and, second, applying acetonitrile. The precipitate on the filter (polypropylene, 0.45 μm pore size) was redispersed again in acetonitrile and further stored there (Figure 1b). The schematics of side and top views in Figures 1c and 1d demonstrate a layered crystalline structure of initial CaSiGe with Ca atoms distributed between SiGe layers, and after removing Ca^2+^, a few-layer SiGe itself.

**Figure 2:**
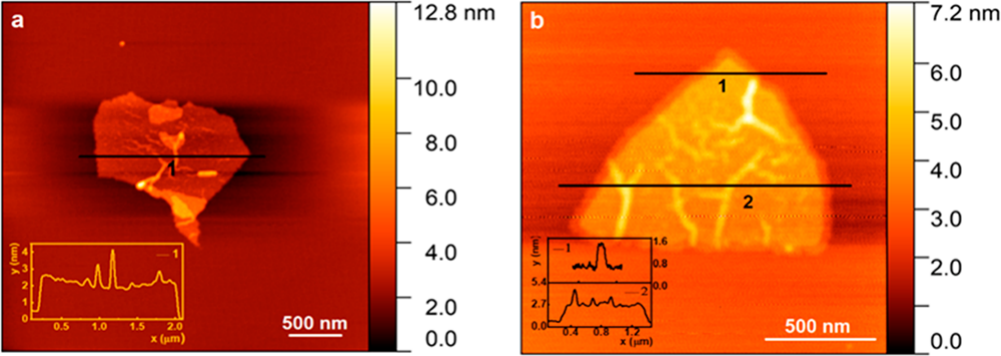
Topological characterization of the electrochemically exfoliated siligene represented by the AFM images and their corresponding height profiles (a, b).

**Figure 3:**
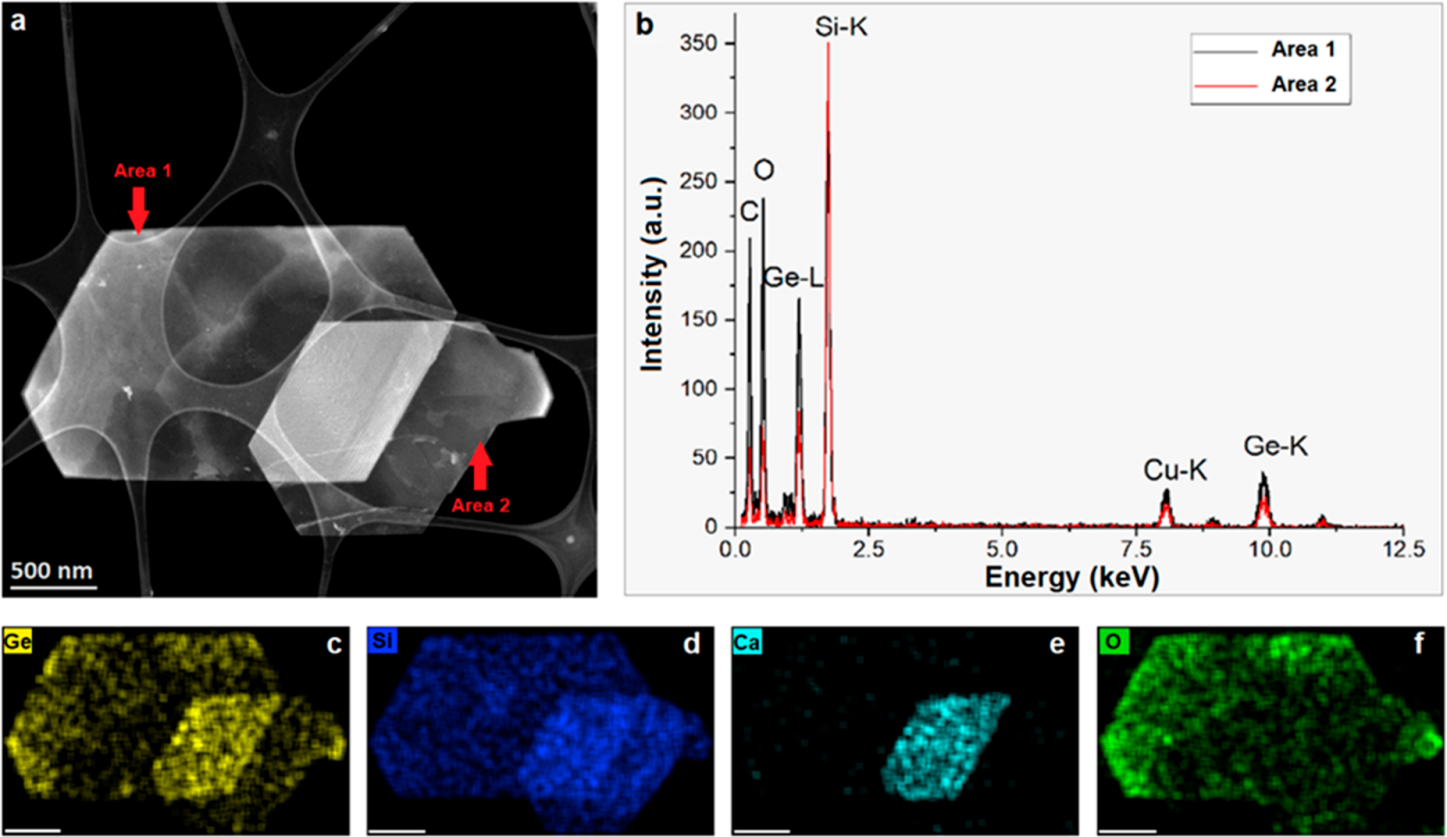
HR-STEM_HAADF image of two flakes of siligene (a); EDS spectra were obtained from different siligene flake areas (b); HR-STEM-EDS maps showing the elemental distribution of Ge (c), Si (d), Ca (e), and O (f) in siligene, respectively. The scale bar of (c)–(f) corresponds to 500 nm.

**Figure 4:**
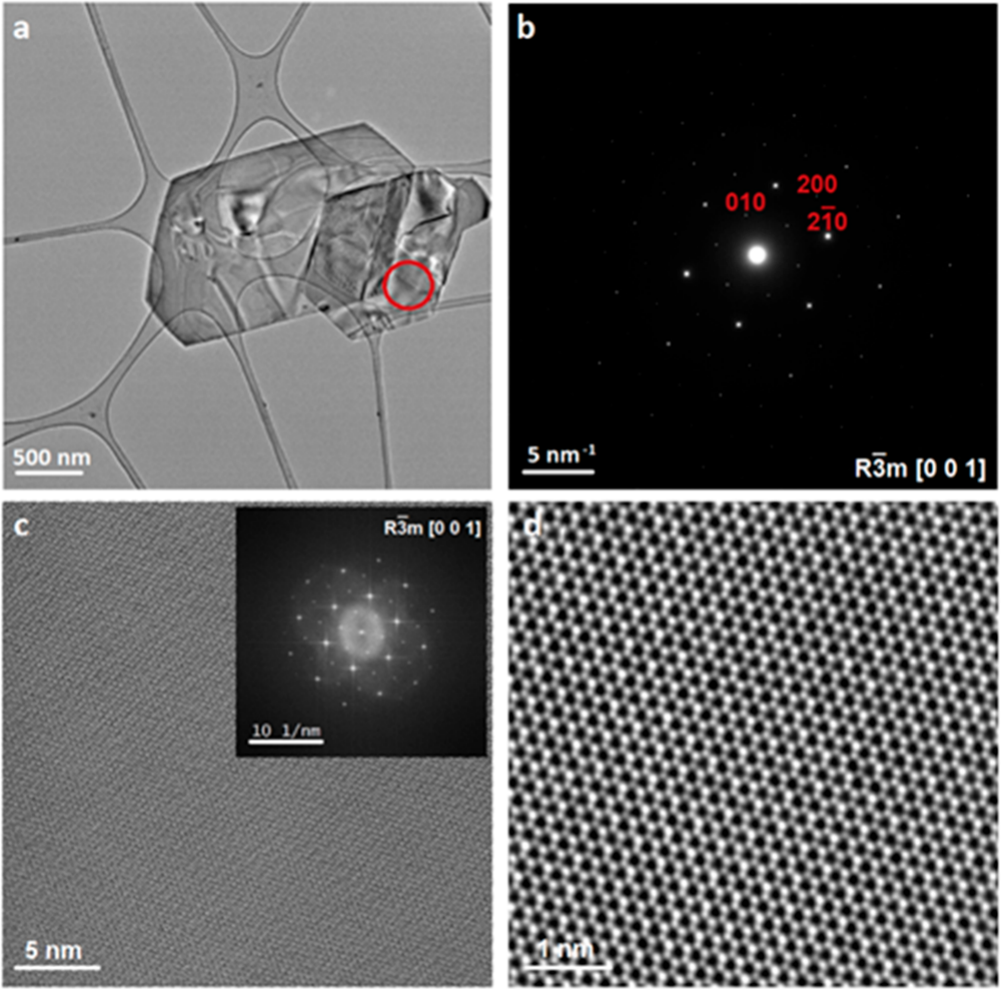
Low-magnification TEM image (a); the red circle highlights the area used to acquire the SAED pattern (b). In the HR-TEM micrograph (c), the inset shows the corresponding FFT pattern. The filtered HR-TEM micrograph (d).

Detailed morphological, structural, and chemical analyses of the exfoliated siligene were revealed by several microscopy techniques, including atomic force microscopy (AFM) and aberration-corrected TEM microscopies. The results of AFM analysis and low-magnifications TEM image of a few-layer electrochemically exfoliated SiGe are shown in Figures 2, S2, and 3a. Both methods confirm that the surface of SiGe flakes is relatively uniform, slightly wrinkled, and contamination-free; the shape of each flake is well-defined, and the lateral size of the flakes is typically between 1 and 2 μm. The low thickness of the SiGe flakes can be clearly appreciated from the (S)TEM images. In particular, the flakes are thin enough to show the presence of the carbon membrane below in both TEM and STEM images (Figures 3a and 4a). The STEM-HAADF image of two flakes of siligene is represented in Figure 3a. The EDS analyses and the corresponding elemental quantification are shown in Figure 3b and Table S1. The atomic ratio of Si/Ge varies between 3.9 and 7.1 for areas 1 and 2, respectively. The presence of oxygen in the EDS spectra is attributed to the material’s slight oxidation, preferably in the area with a lower Si/Ge ratio. It should be noted that in the preoxidation state Ge atoms are more favorable to the oxygen species rather than Si atoms, whereas oxygen molecules adapt better to the Si surface.^8^ Thus, we can assume that the surface is less oxidized when the structure is dominated by Si atoms. In addition, the presence of Ca (about 13 at. %) can be highlighted at the intersection of the two flakes, probably due to an incomplete exfoliation. To achieve complete etching of Ca, additional optimization of the process and thorough postsynthesis washing are necessary. Figure 4b shows the SAED pattern acquired on a single siligene flake. The single-crystalline nature and the high crystalline quality of the flake can evidently be appreciated from the SAED pattern. It should be noted that the SAED was successfully automatically indexed by using the *R*3*®m* structure of CaSi_2_, in which the Ca atoms were removed from the structure. The fast Fourier transform (FFT) pattern acquired on the same area was also successfully automatically indexed by using the same *R*3̅*m* structure (Figure 4c). Once again, the excellent crystalline quality of the flake is highlighted in both the FFT pattern and the HR-TEM micrograph (Figures 4c and d). Analysis of other flakes showed similar results (Figures S3a and S3b).

**Figure 5:**
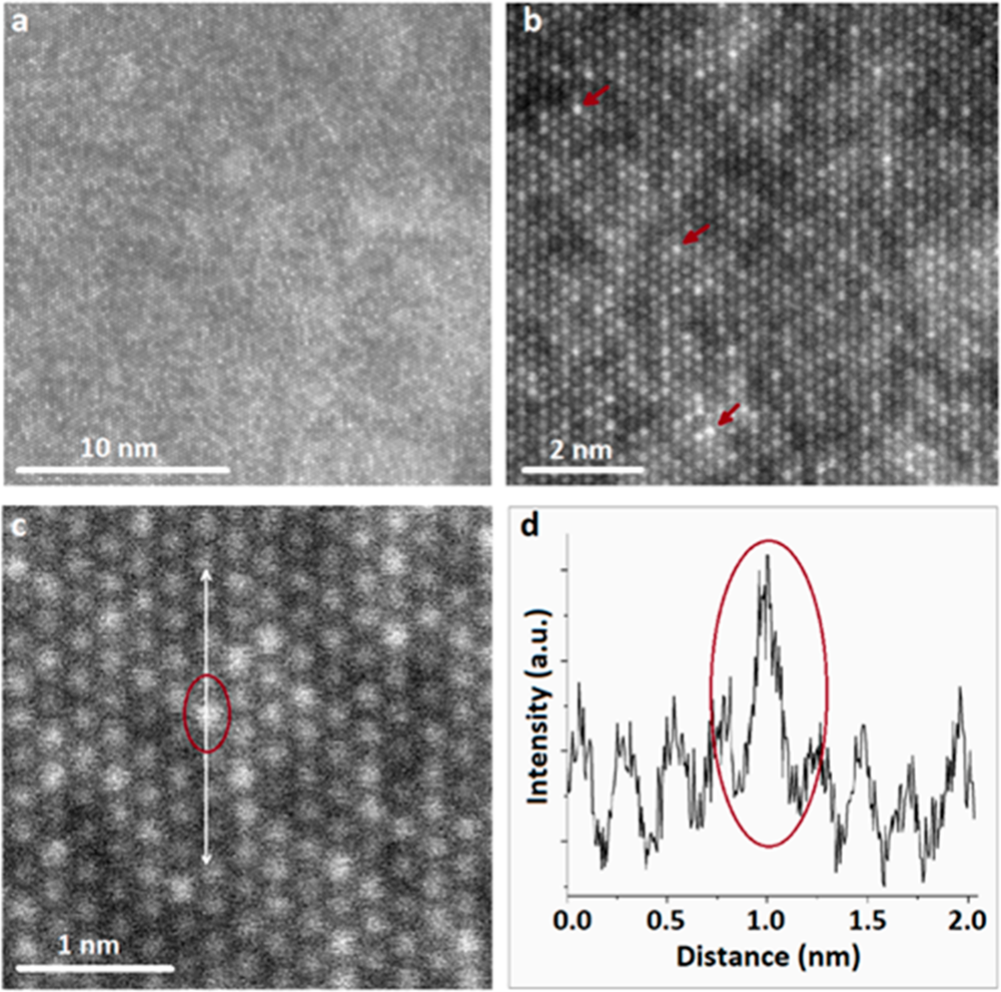
Various magnification HR-STEM HAADF images of the atomic distribution of Si and Ge within the siligene lattice (a–c). The red arrows in (b) highlight the presence of the atomic columns with the highest contrast. The white arrow in (c) highlights the area used to extract the intensity profile (e).

The HR-STEM-HAADF images acquired at different magnifications (Figures 5a–c) confirm the crystallinity of the exfoliated siligene and allow us to identify the Si and Ge distribution within the lattice. The intensity of the HAADF image is dependent on the atomic number and/or thickness; thus atomic columns with the highest intensity can be identified as Ge atoms (Figure 5d). The nonuniform distribution of Si atoms aligns with a previous study on a germanium–silicon solid solution single crystal.^3^ As can be seen from the HR-STEM-HAADF images, the Si and Ge atoms are not agglomerated but randomly dispersed.

**Figure 6:**
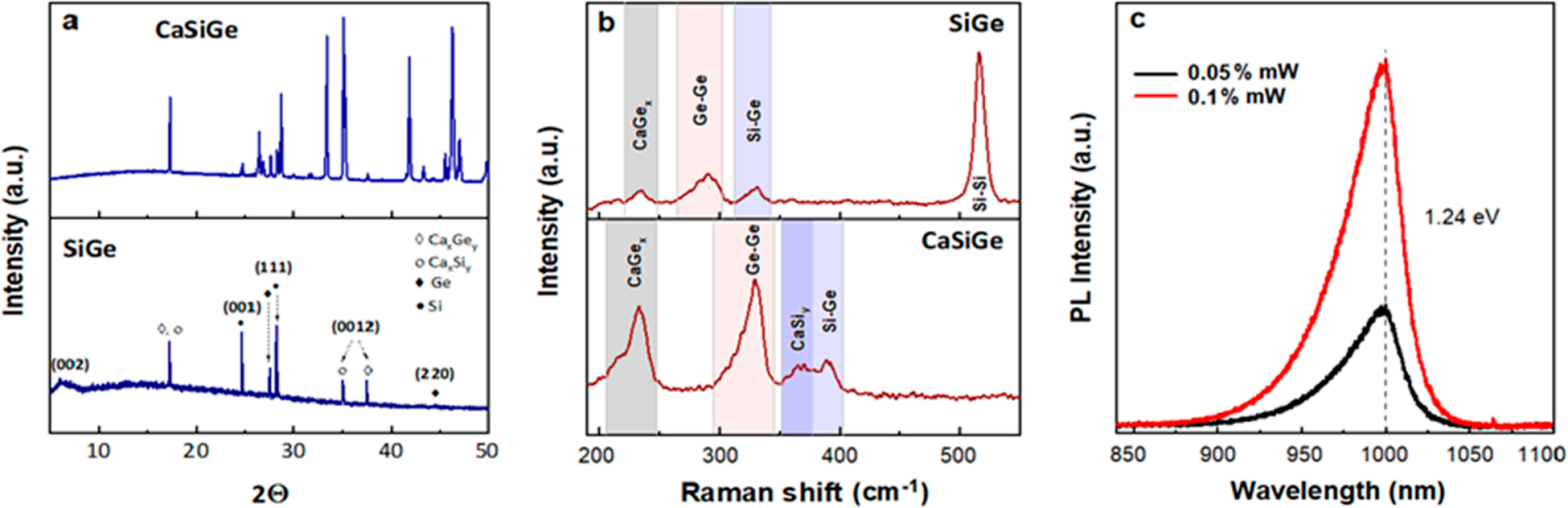
Comparative XRD (a) and Raman spectra (b) of a CaSiGe crystal and exfoliated siligene. Photoluminescence spectra of exfoliated siligene nanosheets (c).

The structure of the exfoliated siligene and its intrinsic crystal CaSiGe were analyzed by XRD (Figure 6a). The XRD pattern possesses relative similarity to hexagonal *R*3̅*m* symmetry when compared with the CaSi_2_ and CaGe_2_^1,9^ and aligns with the SAED patterns (Figure 4b). The low-intensity, broad peak at 6.36° is assigned to the (002) plane of hydrogenated buckled siligene, indicating an interlayer spacing of 6.95 Å. Contrary to previously published results,^10^ the interlayer spacing between nonfunctionalized bilayer silicene and germanene is 4.10 and 3.93 Å, correspondingly. However, the formation of Si/Ge–H bonds increases this distance by about 2.7–3.3 Å and thus agrees with our results referring to successful exfoliation. The diffraction pattern at 24.6° is assigned to (001) plane reflection of silicon; meanwhile, the peaks at 27.4° and 28.1° are assigned to the (111) plane reflections of germanium and silicon, respectively.

The narrow full-width-at-half-maximum of (001) and (111) peaks indicates good crystallinity along the *c*-axis, which is attributed to the layered materials.^11^ The following peaks and their corresponding plane reflection at 17.2° (002), 35.0° (0012), and 37.4° (0012) indicate the presence of the calcium residuals in the forms of CaSi_*x*_Ge_*y*_. A low-intensity peak at 44.4° (220), allocated at the higher reflection angle, was attributed to the planes of germanene, indicating the material’s periodicity and polycrystallinity.

The structure of siligene and its bulk material was further characterized using Raman spectroscopy (Figure 6b). The exfoliated SiGe displays three main Raman features at 235, 290, and 332 cm^–1^, which originate from the out-of-plane A_g_ mode (residuals of CaSi_*x*_Ge_*y*_) and two in-plane E_g_ modes (Ge–Ge and Si–Ge), respectively. When compared to the bulk CaSiGe, we observe a significant redshift of Ge–Ge (by 39 cm^–1^) and Si–Ge bands (by 56 cm^–1^) and a negligible blueshift (by 3 cm^–1^) of the out-of-plane CaGe_*x*_ mode. These shifts are caused by changes in the bond length, which is directly related to the formation of siligene solid solution and disorder in the material’s lattice due to the nonuniform ratio of Si and Ge atoms and hydrogenation or/and oxidation. A high-intensity peak at 516 cm^–1^ indicates a silicene-like buckled structure,^12^ which is induced by the symmetric stretching (E_2g_) of Si–Si bonds within the planar lattice. This peak did not appear in the bulk sample, which verifies its successful transformation in the 2D siligene. In addition, Raman is complementary to the XRD results for the exfoliation of CaSiGe, showing the disappearance of the CaSi_*y*_ Raman mode and referring to the oxidation via Ge centers.

To study the optical properties of exfoliated siligene, we employed UV–vis spectroscopy depicting absorption spectra (Figure S4 a) at room temperature in the range of 300–850 nm (4.13–1.46 eV). The measurement has been carried out several times using different concentrations of the exfoliated few-layer siligene. Since no absorption edge can be observed within this range, the energy bandgap of exfoliated siligene should be below 1.46 eV. We performed a room-temperature PL measurement using an excitation laser with a 532 nm wavelength (Figures 6c, S4b) to further investigate the optical properties of siligene, namely, the optical bandgap that should be approximately similar to the energy bandgap. It has to be noted that as theoretically predicted, the SiGe monolayer demonstrates a direct bandgap of 0.015 eV similar to silicene and germanene.^6^ The density of states study demonstrates the equal contribution of the Si and Ge atoms to the Dirac cone electronic dispersion and refers to a metallic nature in a bilayer form due to its buckled structure. In addition, the estimated bandgap of other SiGe-based derivatives such as siligane (HSiGeH, fully hydrogenated) and siligone (HSiGe, semihydrogenated) varies. For instance, a 2.05 eV/1.85 eV direct bandgap was predicted for a monolayer/bilayer semiconductor HSiGeH, and a 0.62 eV/1.20 eV direct bandgap was calculated for a magnetic/nonmagnetic semiconductor of monolayer/bilayer HSiGe, correspondingly. Based on the above, we assume that electrochemically exfoliated few-layer siligene possesses rather semiconductor than metallic properties, which are caused by interlayer hydrogenation.^13^ This is consistent with the results obtained by Raman, XPS, and our previous study on edge-hydrogenated germanene.^9^

**Figure 7:**
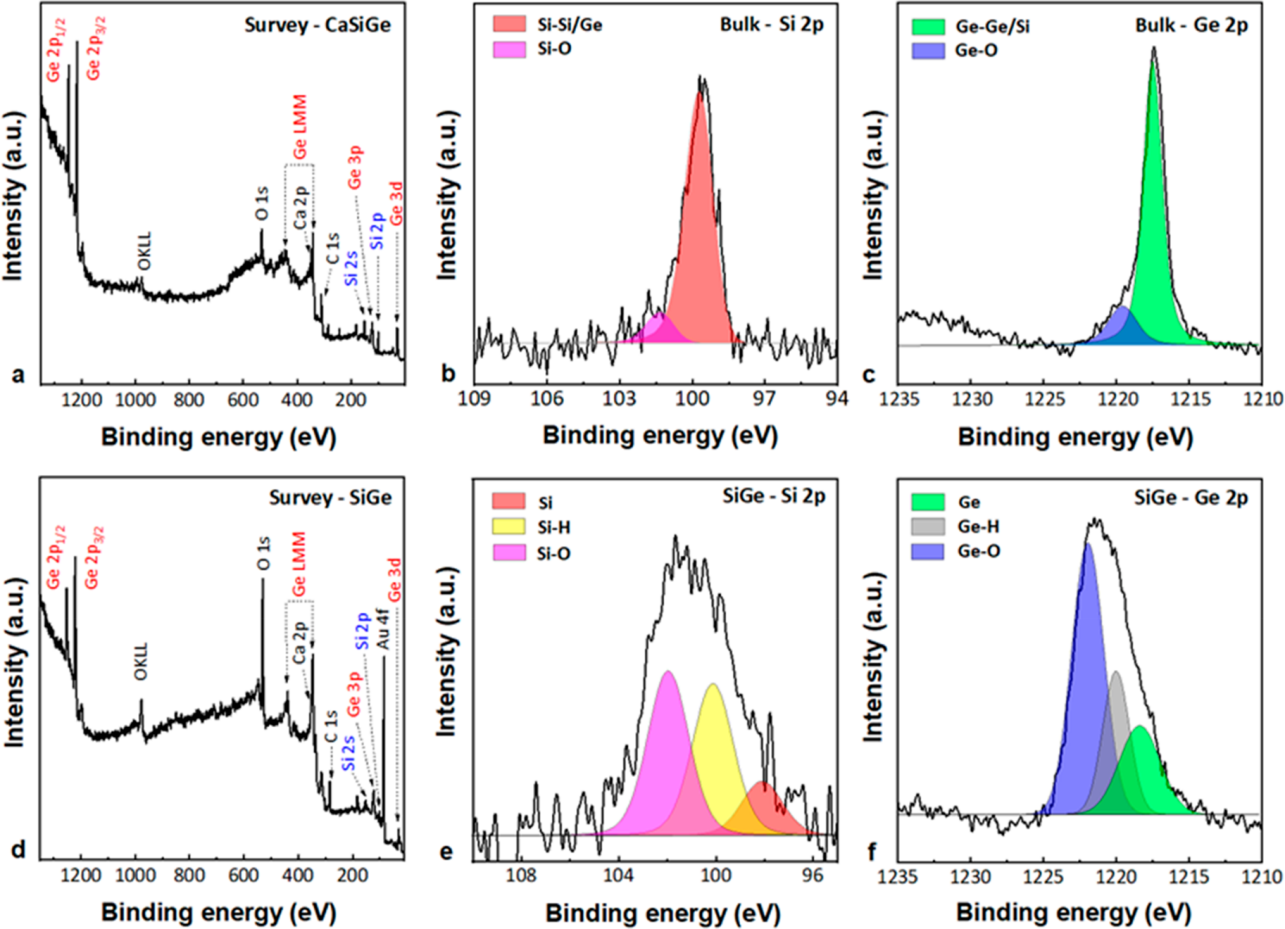
Chemical states of the bonded elements on the surface of bulk CaSiGe and its exfoliated form siligene. XPS survey spectra of bulk crystal (a) and exfoliated sample (d). High-resolution spectra of Si 2p (b, e) and Ge 2p (c, f) states depicted from bulk CaSiGe and exfoliated siligene, correspondingly.

Chemical analysis of electrochemically exfoliated SiGe and its bulk precursor CaSiGe was performed by XPS (Figure 7). The elemental composition of samples shown in wide-scan survey spectra (Figures 7a and 7d) demonstrates the presence of Si, Ge, C, O, Ca, and Au. The peak at 84.1 eV is assigned to Au 4f and happens to appear because we use a golden substrate for the XPS measurements; meanwhile, the peaks at 284.3 eV (C 1s) and 530.9 eV (O 1s) indicate adsorbed species on the surface and oxidation. The low-intensity peak at 350.81 eV displays the Ca 2p region that overlaps with the Ge LMM peak at 346.48 eV for Ge-based samples obtained from Zintl phase precursors. The core-level spectrum of Si 2p for bulk and exfoliated siligene is shown in Figure 7b and Figure 7e, respectively. The Si atom core peak of exfoliated material was deconvoluted into three states at 98.5 eV (14.5%, red area), 100.0 eV (41%, yellow area), and 102.1 eV (44.5%, purple area), corresponding to Si–Si/Ge, Si–H, and Si–O bonding states. In comparison to the Si 2p chemical states of the initial bulk crystal, some unreacted elemental silicon is observable, and Si exhibited higher surface oxidation, which is normal after the exfoliation. The Ge 2p spectra for bulk (Figure 7c) and exfoliated samples (Figure 7f) were fit with several peaks referring to elemental germanium, Ge–H, and Ge–O forms. The Ge 2p peak of bulk CaSiGe was deconvoluted in two areas assigned to elemental germanium at 1218.1 eV (82%, light green area) and the oxide form GeO_*x*_ at 1220.2 eV (18%, violet area). For the exfoliated siligene three areas of Ge 2p peak were attributed to the elemental germanium at 1218.23 eV (15.4%, light green area), Ge–H at 1219.86 eV (36.5%, gray area), and Ge–O at 1221.81 eV (48.1%, violet area). The results demonstrate the material’s tendency toward oxidation and surface termination with low-level hydrogenation, accordingly.^9^

### Siligene-Integrated Anode for Lithium-Ion Batteries

The electrochemical performance of silicene (a 2D form of Si) and germanene (a 2D form of Ge) as electrode materials for LIBs has been demonstrated by their good specific capacity^14,15^ and cyclic stability over up to 1800 charge–discharge cycles.^16^ It is expected that both silicene and germanene will exhibit a similar performance due to their analogous structural and electronic properties. However, due to the decomposition of the electrolyte and the formation of a solid electrolyte interphase (SEI) and, thus, the material’s low electrical conductivity, silicene-based batteries exhibit increased initial irreversible capacity that affects the life of the batteries. Germanene, on the other hand, possesses a slightly higher electrical conductivity and therefore better electrochemical performance,^17^ but does not exist naturally unfunctionalized and struggles in the oxygen-containing environment.^18^ Bearing in mind the exceptional theoretical prediction of capacity rate and electrochemical potential of these materials in their monoelemental form,^19,20^ we assumed that a single-phase siligene composed of Si and Ge atoms simultaneously can enhance the performance of lithium storage. Here, we studied siligene-enabled LIBs using SiGe as an anode material in two configurations, namely, SiGe-MWCNTs and SiGe-rGOS (see details in Battery Fabrication and Characterization).

**Figure 8:**
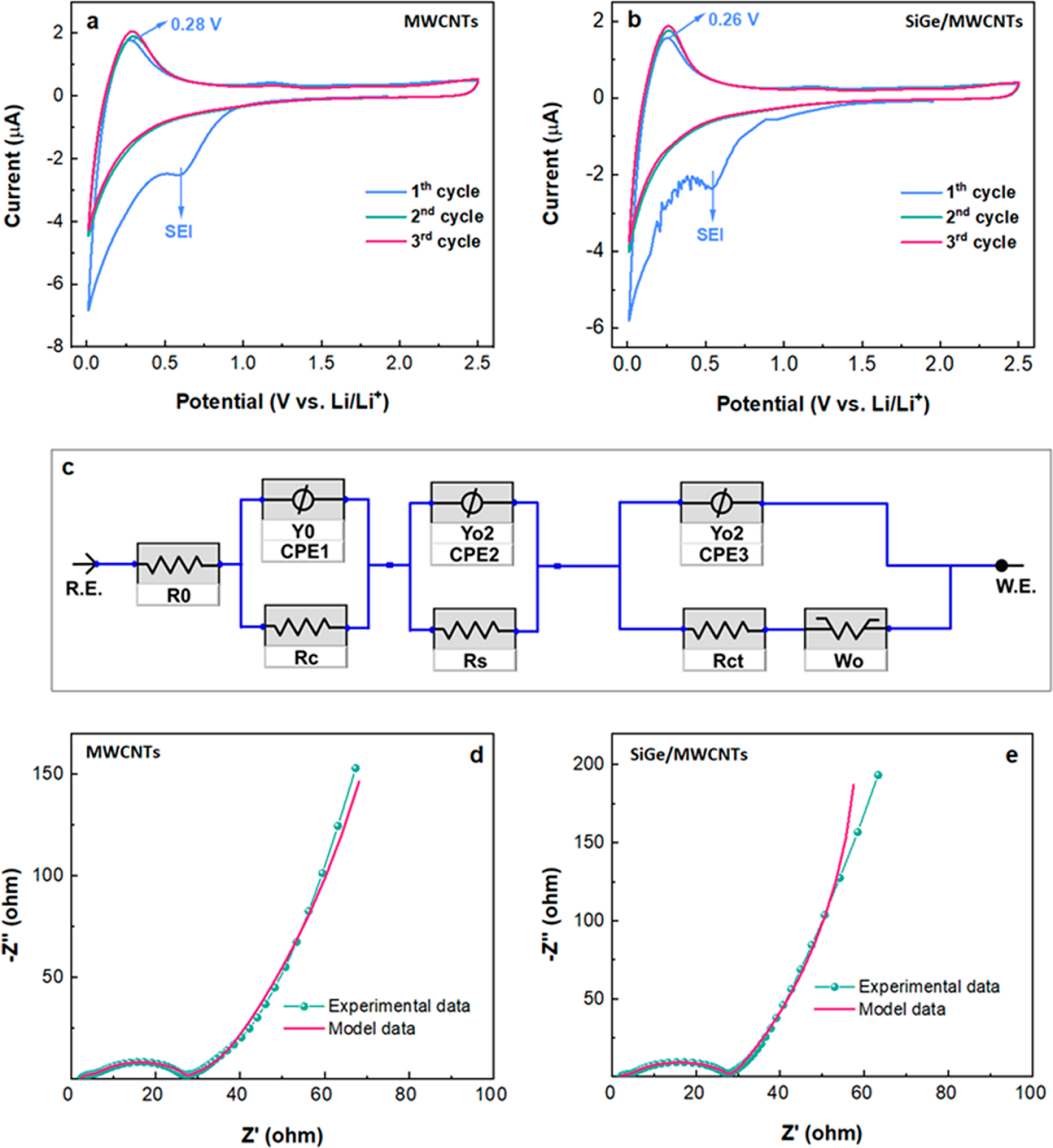
Electrochemical performance of LIBs based on MWCNTs and (SiGe)MWCNTs. Initial 3-cycle cyclic voltammetry curves at 0.2 mV·s^–1^ of the MWCNTs-based battery (a) and SiGe_MWCNTs-based battery (b). The equivalent-circuit diagram (c) and Nyquist plots of MWCNTs- (d) and SiGe_MWCNTs-based batteries (e).

First, to illustrate the lithium storage mechanism, the CV measurements were conducted for initial MWCNT- (control) and SiGe-decorated MWCNT-based (SiGe_MWCNTs) batteries at a scan rate of 0.2 mV s^–1^. Additionally, cycling performance (Figure S5a) and corresponding discharge/charge curves at 100 mA g^–1^ (Figure S5b) have been provided to demonstrate the 2711 mAh g^–1^ initial lithiation capacity of pure SiGe anodes, which agrees well with the 2920 mAh g^–1^ theoretical capacity. As shown in Figures 8a and 8b, both MWCNT and SiGe_MWCNT LIBs displayed a broad peak between 0.8 and 0.4 V during the initial negative scan, contributing to the formation of an SEI. It is known that, on the one hand, the formed SEI layer on the surface of an active material can alleviate the side reaction between active materials and electrolytes.^21^ On the other hand, SEI is nonconductive and can irreversibly consume lithium ions in the electrolyte, resulting in low Coulombic efficiency. The polarization oxidation peak of the MWCNT electrode is at 0.28 V, which is 0.02 V larger than for the SiGe_MWCNT electrode (0.26 V). This can be ascribed to the formation of a thicker nonconductive SEI layer on the MWCNT electrode. During the subsequent cycling, the SEI peaks disappeared, and the observed redox couple at approximately 0 V/0.28 V dominates the following lithiation–delithiation processes.

The electrode kinetics after 3 cycles of CV measurements were further investigated by EIS using a frequency range of 0.01–10^6^ Hz. The equivalent circuit diagram in Figure 8c was selected to illustrate the electrode kinetic behavior, where the *R*_o_ is the ohmic resistance electrode; *R*_c_ is the contact resistance between the electrode and coin cell; *R*_s_ is the resistance of the formed SEI layer; *R*_ct_ is part of charge transfer resistance. Their Nyquist plots and corresponding modeling curves are provided in Figures 8d and 8e, and the calculated results are concluded in Table S2. The following values of *R*_o_, *R*_c_, and *R*_s_ are 2.28, 3.30, and 26.31 Ω for control MWCNTs-based LIB and larger than the corresponding values of 1.96, 2.64, and 12.94 Ω for SiGe_MWCNTs-based LIBs.

The results indicate that the integration of 2D SiGe counterparts into the MWCNT matrix effectively prevents the formation of the SEI layer and, therefore, increases the conductivity and discharge/charge efficiency of the electrode material.

**Figure 9:**
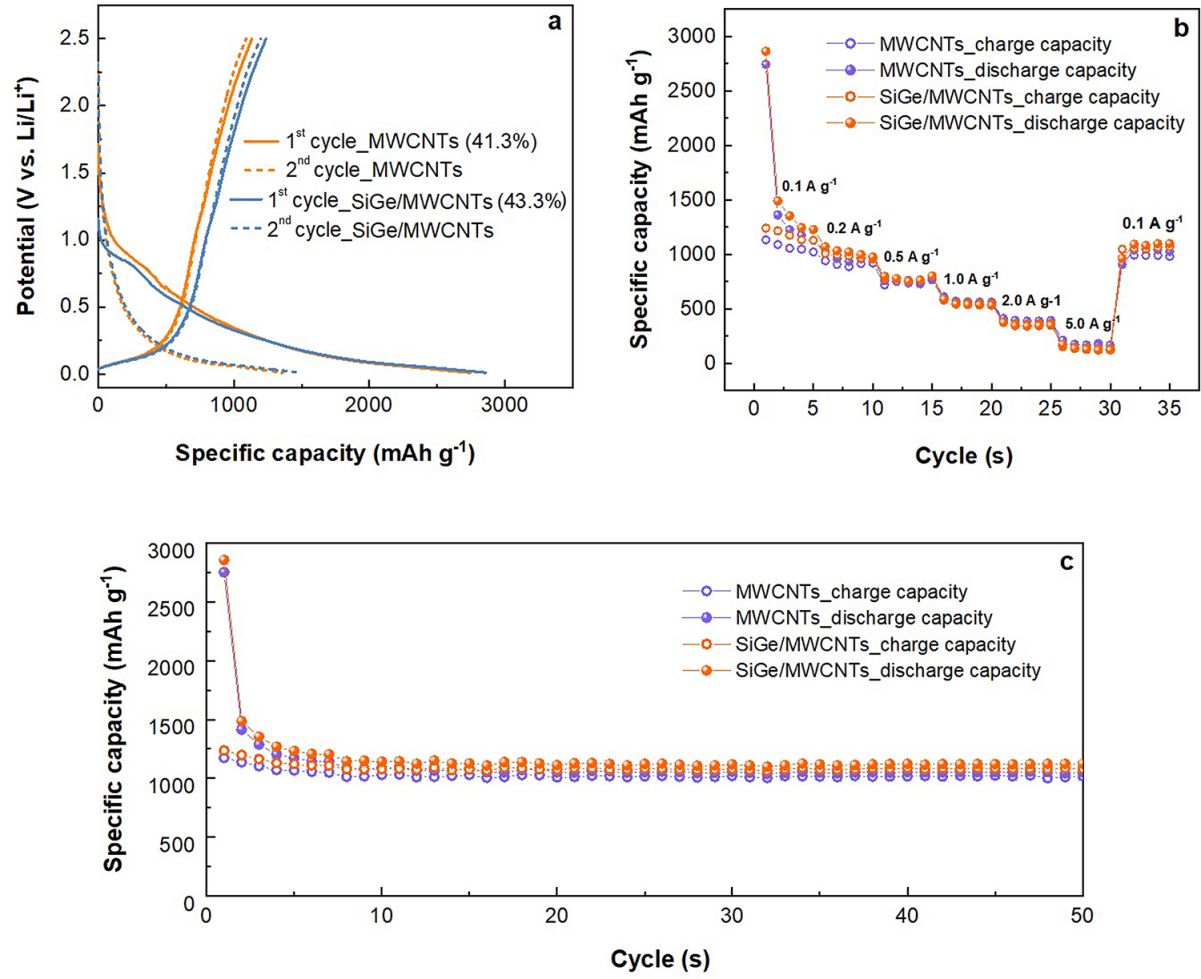
Evaluation of galvanostatic discharge/charge performance of MWCNTs- and SiGe_MWCNTs-integrated LIBs: initial 2 cycles discharge/charge curves at 0.1 A·g^–1^ (a); specific capacity rate performance at different current densities (b); long time cycling performance at 0.1 A·g^–1^ (c).

The galvanostatic discharge/charge analysis of LIBs integrated with both MWCNTs and SiGe_MWCNTs anodes is represented in Figure 9. As shown in Figure 9a, the battery cells deliver an initial discharge/charge specific capacity of 2744.5/1133.8 mAh·g^–1^ with 41.3% Coulombic efficiency for MWCNTs and 2857.0/1237.6 mAh·g^–1^ with 43.3% Coulombic efficiency for SiGe_MWCNTs, respectively. The increased specific capacity originates from the presence of SiGe counterparts, which also enhances the Coulombic efficiency of the battery due to the thinner SEI. In addition, the SEM image comparison (Figure S6) of the electrode thickness expansion rate of the MWCNTs (Figures S6a and S6b) with and without SiGe added during initial discharge to 0.5 V shows a slight decrease in the expansion after adding SiGe, indicating higher resistance to pulverization of SiGe_MWCNTs (Figures S6c and S6d) structure. The rate performance measurements of fabricated LIBs were conducted at a current density rising from 0.1 to 5.0 A g^–1^, which was then restored to 0.1 A·g^–1^. Figure 9b exhibits a higher specific capacity of SiGe_MWCNTs-based LIB at a lower current density for 0.1 to 0.5 A·g^–1^. However, further increment of current density from 1.0 to 5.0 A·g^–1^ leads to a better electrochemical performance of the MWCNTs-based LIB (higher specific capacity), which might be attributed to the high intrinsic conductivity of unmodified MWCNTs. The long-term cycling performance of prepared electrodes was estimated at 0.1 A·g^–1^ during 50 cycles (Figure 9c), demonstrating insignificant decay of the specific capacity for both types of batteries. After the initial activation, both electrodes start exhibiting rather stable capacities from the 10th cycle, namely, 1050.0 mAh·g^–1^ for MWCNTs- and 1145.0 mAh·g^–1^ for SiGe_MWCNTs-based LIBs. The increased capacity rate for the SiGe-integrated LIB, which is 100 mAh·g^–1^ higher, refers to a synergistic effect of anode components. It has to be noted that the most successful combination of electrode materials for Li^+^ storage is based on ion-conductive one-dimensional (1D) paths and/or 2D planes in layered or crystalline structures.^22^ Their electrochemistry is based on the low-volume expansion and contraction that provides advanced mechanical and electrochemical stability during long-term exploitation.

In addition, we compared the electrochemical performance of an LIB composed of (SiGe)rGOS (Figure S7). As shown in Figure S7a, the formation of the SEI layer was assigned to the peaks at around 0.5 V for both rGOS and SiGe_rGOS with twice higher potential in comparison to (SiGe)MWCNTs-based LIBs (Figure S7b). This is due to a stronger physical attraction between 2D siligene layers and 1D MWCNTs’ net structure. At the initially negative scan range of CV from 0.8 to 0.5 V, the rGOS-based LIB demonstrates a more pronounced peak than the SiGe_rGOS-based LIB, indicating the suppression of SEI formation and thus a positive effect of siligene introduction to the rGOS matrix. This tendency agrees with the LIBs composed of (SiGe)MWCNTs and concludes that the introduction of 2D counterparts such as siligene can prevent the formation of the SEI layer and therefore enhance the performance of as-fabricated LIBs. Moreover, the following Nyquist plots of rGOS and GeSi_rGOS in Figure S7c display the decompressed semicircles, in which the size of the semicircle is directly proportional to the value of the resistance. The results also indicate a decrease in the impedance of the SEI layer after rGOS matrix decoration with siligene.

As expected, both the (SiGe)MWCNTs and (SiGe)rGOS anode-based LIBs behave very similarly, while (SiGe)MWCNTs-based LIBs can achieve a better electrochemical performance compared with (SiGe)rGOS anode-based LIBs. The corresponding batteries exhibit a very low polarization that is manifested by their good cycling stability and decreasing SEI level right after the first discharge/charge cycle. This will lead to no-shuttle reactions that would prevent the continuation of the oxidation–redox (lithiation–delithiation) processes and therefore lead to a reversible capacity of batteries.

## Conclusions

In summary, we have demonstrated a controlled electrochemical exfoliation of a few-layer siligene using Zintl phase Ca_1.0_Si_1.0_Ge_1.0_ as a precursor. The successful synthesis was based on the decalcification–intercalation mechanism in a nonaqueous environment, revealing high-quality and high-uniformity 2D SiGe nanosheets with low-level hydrogenation. The obtained siligene is composed of ultrathin micrometer lateral size flakes with mono- or few-layer thickness and excellent crystallinity. In addition, proposed anodes based on the exfoliated siligene for lithium-ion batteries have been explored. To be specific, the SiGe-integrated LIB exhibits a 10% increment of the specific capacity that aligns and/or prevails over the theoretical capacities of double-layer silicene/germanene. Both SiGe_rGOS- and SiGe_MWCNTs-based LIBs display similar electrochemical behavior such as low polarization, good cycling stability, and decay of the SEI level after the first galvanostatic discharge/charge cycle. As suggested, nanostructuring of microscale electrode materials is considered to be an effective approach to increase the electrochemical performance of the corresponding LIBs. Therefore, this study will bring valuable insights into fundamental research of materials chemistry and help to further address the remaining challenges for the practical application of 2D Si/Ge-based (nano)structures.

## Experimental Section

### Materials and Methods

#### Materials

The crystals of Ca_1.0_Si_1.0_Ge_1.0_ were synthesized by direct reaction of elements in quartz ampoule with alumina crucible. The initial high-purity 99.999% germanium, 99.998% silicon, and 99.9% calcium were purchased from Strem and Alfa Aesar, Germany. Tetrabutylammonium perchlorate was obtained from Sigma-Aldrich, Czech Republic. Acetonitrile was obtained from LachNer, Czech Republic. Acetic acid (p.a. purity) was purchased from PENTA, Czech Republic. *N*-Methyl-2-pyrrolidone (NMP) was delivered from Merck. Multiwalled carbon nanotubes were obtained from BASF, and graphene oxide (GO) was made by the Tour method. The electrolyte LiPF_6_ in the mixture of ethylene carbonate (EC) and dimethyl carbonate (DMC) was purchased from Merck.

#### Methods

Topological and structural analysis of the materials was performed by using AFM (NT-MDT Spectrum Instruments), SEM (Tescan Lyra dual microscope), and XRD (Bruker D8). Aberration-corrected transmission electron microscopy analyses, including high-resolution (scanning) TEM imaging, EDS, and SAED, were performed by using an FEI Titan Cubed Themis microscope, which was operated at 80 kV. The Themis is equipped with a double Cs aberration corrector, a monochromator, an X-FEG gun, a super EDS detector, and an ultra-high-resolution energy filter (Gatan Quantum ERS), which allows for working in dual-EELS mode. HR-STEM imaging was performed by using high-angle annular dark-field (HAADF) and annular dark-field (ADF) detectors. Analyses of SAED and FFT patterns were performed by using the atomsk and jems software.^23^ The optical properties of the bulk and exfoliated samples were analyzed by Raman, photoluminescence (both Renishaw inVia), and ultraviolet–visible spectroscopies (LAMBDA 850, Perki-nElmer, USA). XPS (Phoibos 100 – SPECS) was used to study the chemical composition of bulk and exfoliated materials. The electrochemical performance of siligene-integrated LIBs was performed by CV and EIS, both using Gamry Interphase 1010 E (Warminster, USA). Detailed characteristics of the methods and sample preparation are described in Supporting Information (Experiment Details).

### Synthesis of CaSiGe

The synthesis of Ca_1.0_Si_1.0_Ge_1.0_ crystals was carried out in a quartz glass ampule with an aluminum oxide liner. In the ampule were placed calcium granules, germanium, and silicon powder corresponding to 25 g Zintl phase with the composition Ca_1.0_Si_1.0_Ge_1.0_. Calcium excess was used to compensate for losses from the reaction with the ampule and liner. The ampule was evacuated on the base pressure of 1 × 10^–5^ mbar and melt sealed. The ampule was heated at 1100 °C for 1 h and cooled to room temperature using a cooling rate of 0.5 °C·min^–1^. The formed Zintl phase was mechanically removed from the ampule and stored in an argon atmosphere glovebox.

### Electrochemical Exfoliation of the Si_*x*_Ge_*y*_ Nanosheets

Electrochemical exfoliation of 2D SiGe was performed in a two-compartment electrochemical cell setup of 50 mL volume constructed of the counter and working electrodes. The initial CaSiGe crystal was fixed in a Teflon holder with a platinum electrode that served as a working electrode (anode). As the counter electrode (cathode), we used a platinum plate of 1 × 2 cm^2^ size. The electrochemical cell was filled with an electrolyte composed of 0.03 M TBAClO_4_ in acetonitrile and was purged with argon during the whole exfoliation process. The procedure involves three main voltage steps (−2, −2.87, and −3.8 V) and takes 2 min for the first two steps and at least 5 h for the last one. After the termination of the procedure, exfoliated material was transferred to the vial and ultrasonicated in the initial electrolyte for 30 min. It was followed by washing the samples, first, in 0.01 N acetic acid and, second, in acetonitrile using the vacuum filtration method. A filtered cake of exfoliated material was redispersed in acetonitrile after a few minutes of ultrasonication and stored in an oxygen-free environment for further analysis.

### Battery Fabrication and Characterization

#### Preparation of SiGe-MWCNTs Electrode

The commercial MWCNT powder of 1.0 g was dispersed in 100 mL of NMP and further sonicated for 3 h, keeping the dispersion in the ice bath. To obtain the free-standing MWCNT films, the dispersion was vacuum-filtrated on the PTFE membrane and dried in a vacuum for 12 h. The as-prepared MWCNT films were punched into a 10 mm diameter disk as the support for SiGe nanosheets. Further, the round MWCNT-based support layer was dipped into high-concentration SiGe (around 15 mg mL^–1^) in an acetonitrile medium, followed by 30 min of continuous stirring for better interaction between MWCNTs and SiGe nanosheets. Finally, the SiGe-MWCNTs composite electrodes were dried at 60 °C in the Ar-filled glovebox for further battery assembly. The loading amount of SiGe on MWCNT electrodes was near 5 wt %. In addition, the initial free-standing MWCNT support without loading of SiGe was set as the control experiment.

#### Preparation of SiGe-rGOS Electrode

The samples of rGOS were prepared using hydrothermal synthesis. For that, the well-dispersed graphene oxide (10 mL of 5 mg mL^–1^ in water) was sealed into the 25 mL Teflon-lined stainless vessel. The vessel was placed into the autoclave, then sealed in the air and heated at 160 °C for 12 h. Obtained rGOS was released from the vessel and dipped into the SiGe in an acetonitrile dispersion (around 15 mg mL^–1^), followed by 30 min of stirring for better interaction between rGOS and SiGe nanosheets. Then, the SiGe-integrated rGOS was dried at 60 °C for 3 h in an Ar-filled glovebox. The mass ratio of rGOS:SiGe in the final composite was 85%:15%, correspondingly.

#### Lithium-Ion Battery Assembly and Performance Characterization

The prepared electrodes, both SiGe-MWCNTs and SiGe-rGOS, were assembled into a CR2032 coin cell using lithium foil as a counter electrode and polypropylene membrane (Celgard 2400) as a separator. The mixture of 1 M LiPF_6_ in the ethylene carbonate and dimethyl carbonate (1:1, v/v) served as an electrolyte. The galvanostatic charge/discharge measurements were conducted on a Neware battery test system (Neware BTX 7.6, Shenzhen, China) in a fixed potential window from 0.0 to 2.5 V (vs Li^+^/Li). The CV and EIS were employed to characterize the electrochemical performance of as-fabricated batteries; the measurements were assisted with a Gamry Interface 1010 E electrochemical workstation.

## Supporting Information Available

Experiment details of atomic force microscopy, scanning electron microscopy, high-resolution transmission electron microscopy–selected area electron diffraction, high-resolution scanning transmission electron microscopy–high-angle annular dark-field, X-ray diffractometry, Raman spectroscopy, photoluminescence spectroscopy, ultraviolet–visible spectroscopy, X-ray photoelectron spectroscopy, cyclic voltammetry, electrochemical impedance spectroscopy; Figures of (S1) the experimental LSV, (S2) AFM images, (S3) UV–vis absorption spectra, and (S4) cyclic voltammetry curves; Tables: (S1) HR-STEM-EDS analysis of the elemental distribution of siligene in areas 1 and 2; (S2) values from modeled Nyquist plots of MWCNTs- and SiGe_MWCNTs-based batteries (PDF)

## Notes

A preprint was published: Evgeniya Kovalska, Bing Wu, Liping Liao, Vlastimil Mazanek, Ivo Marek, Luc Lajaunie, Zdenek Sofer. Electrochemical exfoliation of two-dimensional siligene Si_*x*_Ge_*y*_; material characterization and perspectives for lithium-ion storage. 10.26434/chemrxiv-2022-pt6xb. ChemRxiv. Cambridge: Cambridge Open Engage; https://chemrxiv.org/engage/chemrxiv/article-details/62fe5548d858fb72be52f621, August 19, 2022.
