# Supplementary material for: Electrochemical Decalcification–Exfoliation of Two-Dimensional Siligene, SixGey: Material Characterization and Perspectives for Lithium-Ion Storage
Source: ACS Nano. 2023 Jun 7;17(12):11374–83. doi: 10.1021/acsnano.3c00658 (PMC10311597; doi:10.1021/acsnano.3c00658)
Supplement: Supplementary file 1 — nn3c00658_si_001.pdf [file nn3c00658_si_001.pdf]

Supporting Information

Electrochemical decalcification-exfoliation of two-dimensional siligene Si_xGe_y; material characterization and perspectives for lithium-ion storage

Evgeniya Kovalska,[1,2] Bing Wu,[1] Liping Liao,[1] Vlastimil Mazanek,[1] Jan Luxa,[1] Ivo Marek,[1] Luc Lajaunie,[3,4] Zdenek Sofer[1*]*

[1]Department of Inorganic Chemistry, University of Chemistry and Technology Prague, Technická 5, 166 28 Prague 6, Czech Republic

[2]Department of Engineering, Faculty of Environment, Science and Economy, University of Exeter, Exeter, EX4 4QF, United Kingdom

[3]Departamento de Ciencia de los Materiales e Ingeniería Metalúrgica y Química Inorgánica, Facultad de Ciencias, Universidad de Cádiz, Campus Río San Pedro S/N, Puerto Real, Cádiz, 11510 Spain

[4]Instituto Universitario de Investigación de Microscopía Electrónica y Materiales (IMEYMAT), Universidad de Cá-diz, Campus Río San Pedro S/N, Puerto Real, Cádiz, 11510 Spain

Contents:

(I) Experiment Details

- Atomic Force Microscopy
- Scanning Electron Microscopy
- High-Resolution Transmission Electron Microscopy – Selected Area Electron Diffraction
- High-Resolution Scanning Transmission Electron Microscopy – High-Angle Annular Dark-Field
- X-ray diffractometry
- Raman Spectroscopy
- Photoluminescence Spectroscopy
- Ultraviolet-Visible Spectroscopy
- X-ray Photoelectron Spectroscopy

- Cyclic Voltammetry
- Electrochemical Impedance Spectroscopy

(II) Figures

Figure S1. The experimental linear sweep voltammetry (LSV) recordings during electrochemical exfoliation of $Ca_{1.5}Si_{1.0}Ge_{1.0}$ in 0.03 M $TBAClO_4$ in acetonitrile at −3.8 V. The graph of current vs. voltage shows three stages at the following potential −2.0, −2.87, and −3.8 V correspond to the accumulation, decalcification-intercalation, and exfoliation processes. (**a**) The intercalation starting potential is in the range of −2.0 to −2.2 V, and the decalcification starting potential is −2.87 V (corresponds to the reduction potential of Ca^{2+}). The plot of current as a function of time recorded during 1 hour of electrochemical exfoliation of $Ca_{1.5}Si_{1.0}Ge_{1.0}$ (**b**).

Figure S2. AFM images of the electrochemically exfoliated siligene represented by 8 flakes (**a**) which are marked by the numbers 1–8 (**b**), and their corresponding height profiles (**c**).

Figure S3. Morphology of as exfoliated siligene flakes: low-magnification STEM image (a), corresponding HR-TEM micrograph (**b**), the inset shows the FFT pattern; low-magnification TEM image (**c**), corresponding HR-TEM micrograph (**d**), the inset shows the FFT pattern.

Figure S4. UV-Vis absorption spectra (**a**) and estimated band-gap (**b**) of electrochemically exfoliated few-layer siligene in acetonitrile. The band-gap evaluation was based on the results of photoluminescence spectroscopy.

Figure S5. Cycling performance (**a**) and corresponding discharge/charge curves (**b**) of pure siligene at 100 mA g^{-1}.

Figure S6. Comparison of SEM images of the MWCNTs (**a, b**) and SiGe_MWCNTs (**c, d**) electrodes thickness before (**a, c**) and after (**b, d**) expansion rate during initial discharge to 0.5 V.

Figure S7. Initial 3-cycle cyclic voltammetry curves at 0.2 $mV \cdot s^{-1}$ of rGOS electrode (a) and SiGe–rGOS electrode (b). Comparative Nyquist plot diagrams of rGOS and SiGe–rGOS electrodes (c).

(III) Tables

Table S1. HR-STEM-EDS analysis of the elemental distribution of siligene in areas 1 and 2.

Table S2. The values from modelled Nyquist plots of MWCNTs- and SiGe_MWCNTs-based batteries.

(I) Experiment Details

- Atomic Force Microscopy (AFM; NT-MDT Spectrum Instruments)

Morphological analysis of a few-layer siligene was performed by the AFM technique. The measurements were carried out under ambient conditions with a scan rate of 1 Hz and a scan line of 512. The cantilever was working in a tapping mode with a strain constant of 1.5 kN m^{-1} equipped with a standard silicon tip with a curvature radius lower than 10 nm. Samples were prepared following two steps of centrifugation (1000 rpm for 5 min, 8000 rpm for 10 min for supernatant N1), supernatant N2 was then drop-casted on a silicon wafer and dried in the oven (10-15 min at 60 °C).

- Scanning Electron Microscopy (SEM; Tescan Lyra dual microscope)

The morphology analysis of the electrode materials before and after lithiathion was performed by SEM using a field emission gun electron source.

- High-Resolution Transmission Electron Microscopy – Selected Area Electron Diffraction (HRTEM–SAED)

Aberration-corrected transmission electron microscopy analyses, including HR-(S)TEM, energy-dispersive X-ray spectroscopy (EDS) and SAED, were performed by using an FEI Titan Cubed Themis microscope which was operated at 80 kV. The Themis is equipped with a double Cs aberration corrector, a monochromator, an X-FEG gun, a super EDS detector, and an Ultra High-Resolution Energy Filter (Gatan Quantum ERS) which allows for working in Dual-EELS mode.

- High-Resolution Scanning Transmission Electron Microscopy – High-Angle Annular Dark-Field (HRSTEM–HAADF)

HR-STEM imaging was performed by using high-angle annular dark-field (HAADF) and annular dark-field (ADF) detectors. Analyses of SAED and Fast Fourier Transform (FFT) patterns were performed by using the atomsk and jems software.

- X-ray diffractometry (XRD; Bruker D8)

X-ray diffractometry (XRD; Bruker D8) in Bragg–Brentano parafocusing geometry and applying Cu Kα radiation (λ = 0.1540598 nm, U = 40 kV, I = 40 mA). The samples for XRD were prepared by drop-casting the exfoliated materials on a silicon wafer and dried for 20 min in the vacuum oven at 80 °C.

- Raman Spectroscopy (Renishaw inVia)

Raman spectroscopy (Renishaw inVia) is equipped with a charge-coupled device detector and a 532 nm DPSS laser (50 mW). Raman measurements were carried out at ambient conditions employing a 50× objective, a 10 s integration time, and a laser power of 5 mW for a single measurement. The analysis was carried out on the samples placed on a silicon wafer.

- Photoluminescence Spectroscopy (Renishaw inVia)

For PL measurements, the sample was excited with a 532 nm laser applying a laser power of 0.05% and 0.1% mW for a single measurement. The PL signal was collected with the 50× objective and analyzed with the Avantes CCD spectrometer (AvaSpec-ULS2048x64TEC-EVO). The analysis was carried out on the samples placed on a silicon wafer.

- Ultraviolet-Visible Spectroscopy (UV-Vis; LAMBDA 850, PerkinElmer, USA)

UV–vis measurements were carried out using a LAMBDA 850+ UV–vis spectrophotometer (PerkinElmer, USA) with an integrating sphere. Dispersion of the SiGe in acetonitrile was prepared and placed in the quartz cuvette. The absorption spectra were acquired in the range of 300–800 nm.

- X-ray Photoelectron Spectroscopy (XPS; Phoibos 100 – SPECS)

XPS was used to confirm the chemical composition and binding states of bulk and exfoliated samples. XPS spectra were acquired with a monochromatic Al X-ray radiation source (1486.7 eV) and a Phoibos 100 spectrometer (SPECS). The samples were placed from the solution on top of a silicon wafer and dried for 20 min in the oven at 80 °C.

- Cyclic Voltammetry (CV)

The measurements were carried out using an electrochemical workstation (Gmary Interface 1010 E, Warminster, USA). And the whole electrochemical measurements are conducted at room temperature.

- Electrochemical Impedance Spectroscopy (EIS)

The measurements were carried out using an electrochemical workstation (Gmary Interface 1010 E, Warminster, USA). And the whole electrochemical measurements are conducted at room temperature.

(II) Figures

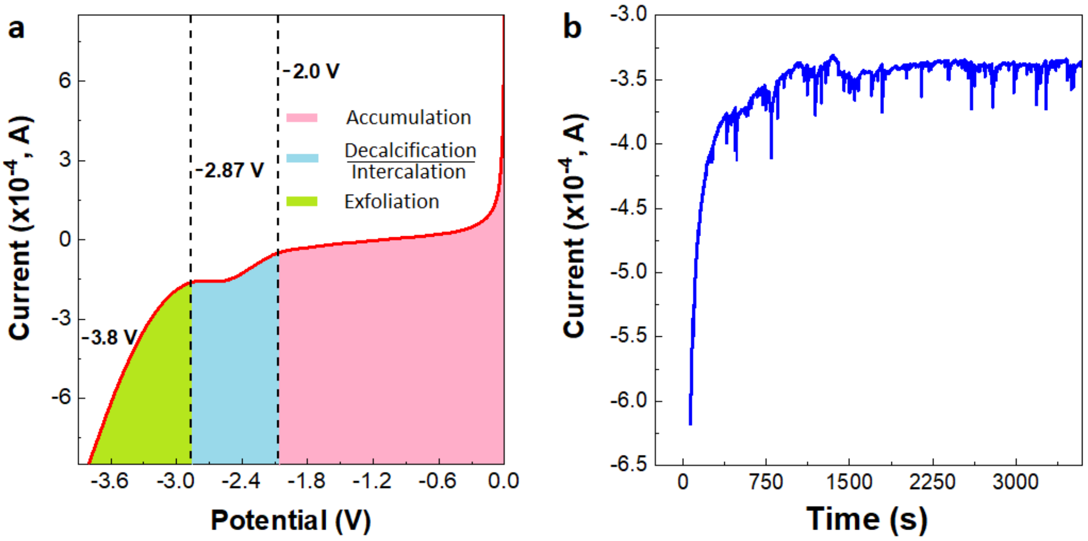

Figure S1. The experimental linear sweep voltammetry (LSV) recordings during electrochemical exfoliation of $Ca_{1.5}Si_{1.0}Ge_{1.0}$ in 0.03 M $TBAClO_4$ in acetonitrile at −3.8 V. The graph of current vs. voltage shows three stages at the following potential −2.0, −2.87, and −3.8 V correspond to the accumulation, decalcification-intercalation, and exfoliation processes. (**a**) The intercalation starting potential is in the range of −2.0 to −2.2 V, and the decalcification starting potential is −2.87 V (corresponds to the reduction potential of Ca^{2+}). The plot of current as a function of time recorded during 1 hour of electrochemical exfoliation of $Ca_{1.5}Si_{1.0}Ge_{1.0}$ (**b**).

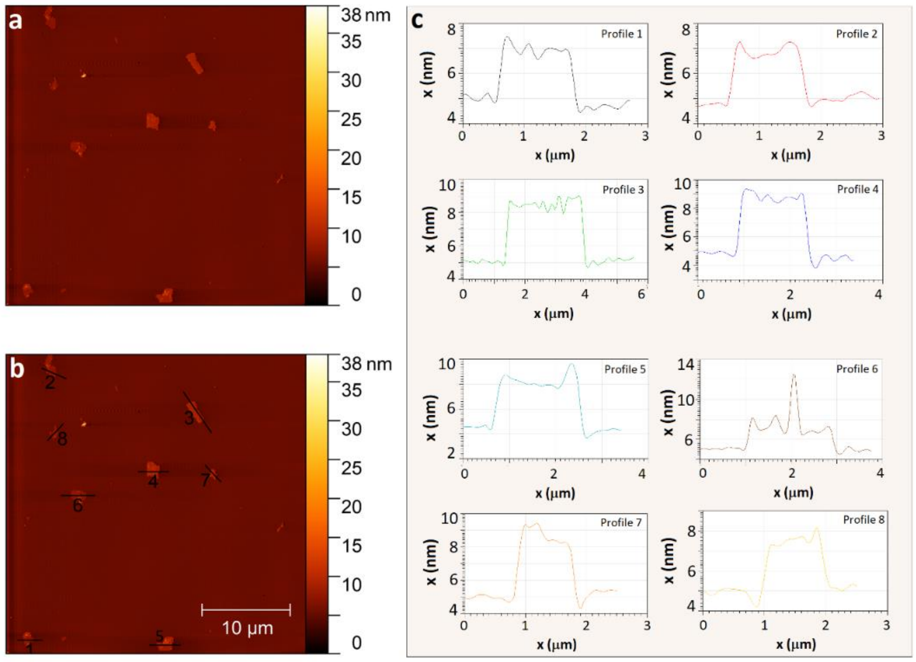

Figure S2. AFM images of the electrochemically exfoliated siligene represented by 8 flakes (**a**) which are marked by the numbers 1–8 (**b**), and their corresponding height profiles (**c**).

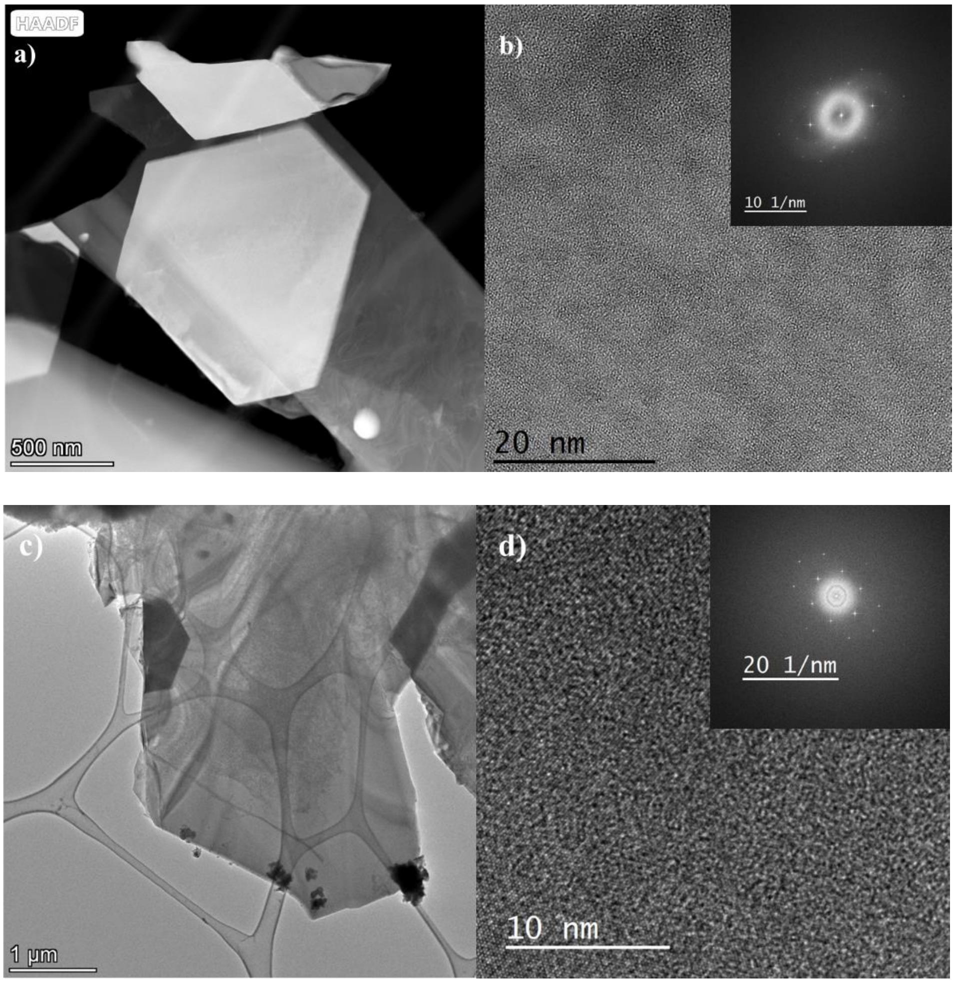

Figure S3. Morphology of as exfoliated siligene flakes: low-magnification STEM image (**a**), corresponding HR-TEM micrograph (**b**), the inset shows the FFT pattern; low-magnification TEM image (**c**), corresponding HR-TEM micrograph (**d**), the inset shows the FFT pattern.

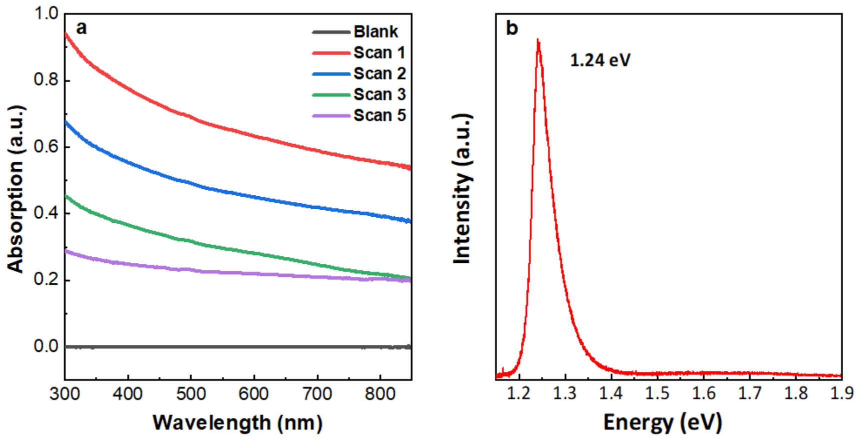

Figure S4. UV-Vis absorption spectra (**a**) and photoluminescence spectrum (**b**) of electrochemically exfoliated few-layer siligene in acetonitrile. The optical band-gap (1.24 eV) evaluation was based on the results of photoluminescence spectroscopy.

The electrochemical performance of pure siligene was measured using electrodes containing 70% pure siligene, 15% PVDF and 15% carbon black. As illustrated in Figure S4 a, the as-prepared siligene exhibits an initial lithiation capacity of 2711 mAh g^{-1}, which is proximal to its theoretical capacity (2920 mAh g^{-1} for $Li_{22}Si_5$ and $Li_{22}Ge_5$).[1-2] However, the initial Coulombic efficiency of pure siligene is only 46%, which is typical for Si and Ge-based materials, arising from the loss of active material due to significant volume variation following lithiation and poor conductivity of the lithiated end products. Consequently, there is a pressing need for improvement methods to enhance the material's Coulombic efficiency in the future. Subsequent assessment of its charge-discharge curve (Figure S4 b) reveals that only a portion of the lithium can be reversibly extracted from the lithiated material during charging, e.g. the oxidation process. Upon increasing the number of cycles, the electrode material becomes activated, and the charging capacity during the oxidation process slightly improves, enabling more lithium to be extracted from the material.

After initial discharging and charging, the battery's performance stabilizes with a capacity close to 1400 mAh g^{-1}, indicating its potential as an anode candidate beyond graphite. Regarding the material capacity, our prepared siligene with a composition of $Si_{0.5}Ge_{0.5}$ exhibits even better results[3] than those reported,[4] with an initial discharge of 2183 mAh g^{-1} and a 10th charge capacity of 1141 mAh g^{-1}.

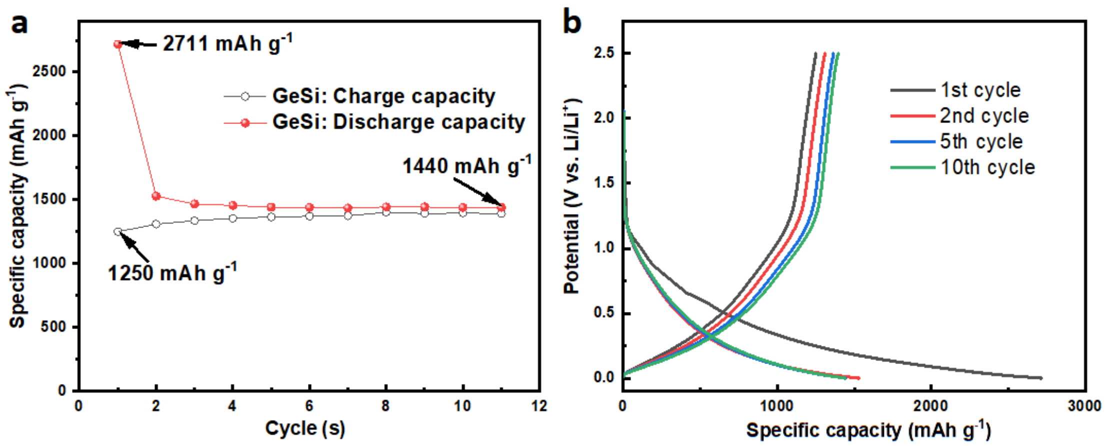

Figure S5. Cycling performance (**a**) and corresponding discharge/charge curves (**b**) of pure siligene at 100 mA g^{-1}.

[1] Im, H. S.; Lim, Y. R.; Cho, Y. J.; Park, J.; Cha, E. H.; Kang, H. S. Germanium and tin selenide nanocrystals for high-capacity lithium ion batteries: Comparative phase conversion of germanium and tin. *The Journal of Physical Chemistry C* **2014,** *118* (38), 21884-21888.

[2] Kwon, T.-W.; Choi, J. W.; Coskun, A. The emerging era of supramolecular polymeric binders in silicon anodes. *Chemical Society Reviews* **2018,** *47* (6), 2145-2164.

[3] Chen, X.; Loaiza, L. C.; Monconduit, L.; Seznec, V. 2D silicon–germanium-layered materials as anodes for li-ion batteries. *ACS Applied Energy Materials* **2021,** *4* (11), 12552-12561.

[4] ACS Appl. Energy Mater. 2021, 4, 11, 12552–12561.

[5] Sannyal, A.; Ahn, Y.; Jang, J. First-principles study on the two-dimensional siligene (2D SiGe) as an anode material of an alkali metal ion battery. *Computational Materials Science* **2019,** *165*, 121-128.

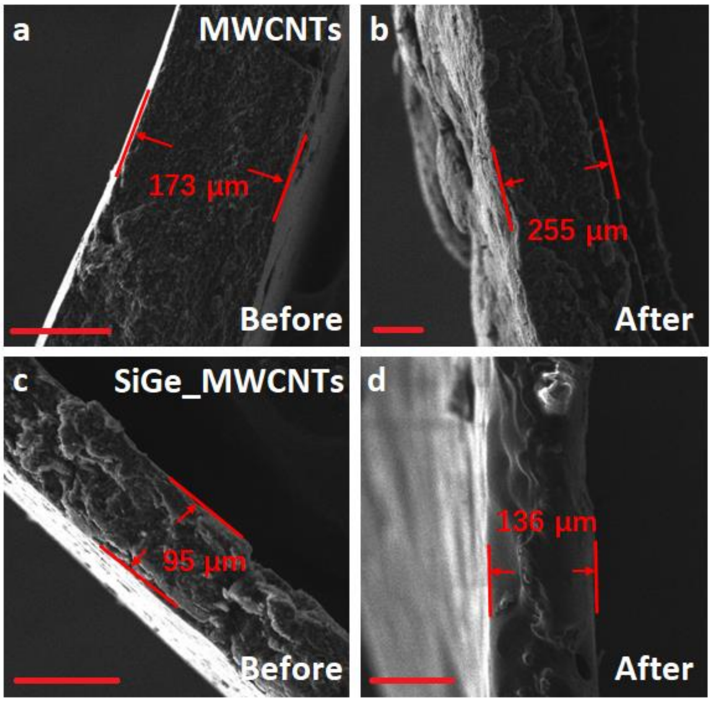

Figure S6. Comparison of SEM images of the MWCNTs (**a, b**) and SiGe_MWCNTs (**c, d**) electrodes thickness before (**a, c**) and after (**b, d**) expansion rate during initial discharge to 0.5 V.

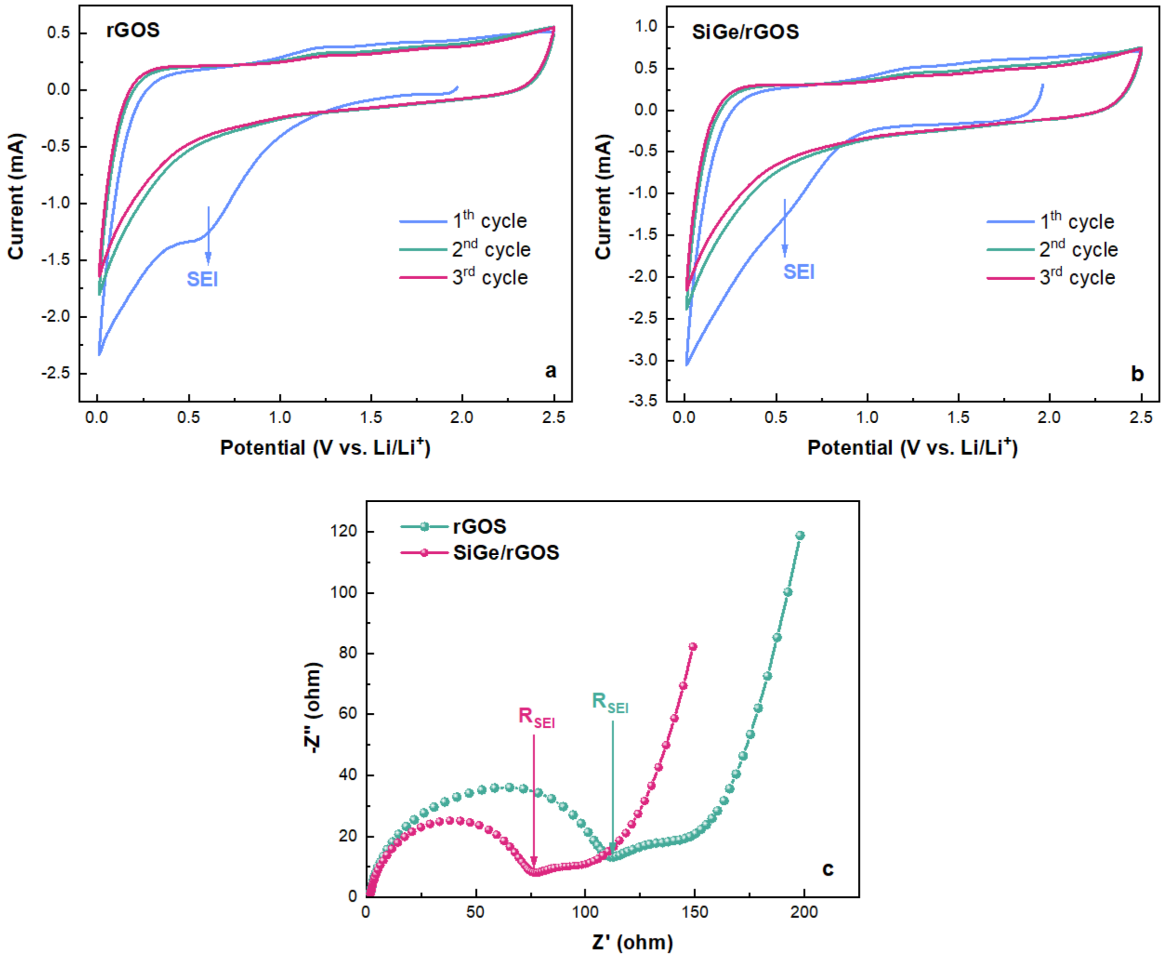

Figure S7. Initial 3-cycle cyclic voltammetry curves at 0.2 mV·s^{-1} of rGOS electrode (**a**) and SiGe–rGOS electrode (**b**). Comparative Nyquist plot diagrams of rGOS and SiGe–rGOS electrodes (**c**).

(III) Tables

Table S1. HR-STEM-EDS analysis of the elemental distribution of siligene in areas #1 and #2.

Z	Element	Family	Atomic Fraction (%)	Atomic Error (%)	Mass Fraction (%)	Mass Error (%)	Fit error (%)
**Area #1 → Si/Ge = 3.91**							
14	Si	K	79.33	6.31	59.95	3.19	0.43
20	Ca	K	0.40	0.08	0.43	0.09	13.86
32	Ge	K	20.27	3.11	39.62	5.61	0.69
**Area #2 → Si/Ge = 7.04**							
14	Si	K	87.56	4.77	73.13	2.70	0.33
32	Ge	K	12.44	1.77	26.87	3.67	0.80

Table S2. The values from modelled Nyquist plots of MWCNTs- and SiGe_MWCNTs-based batteries.

Resistance (Ω)	MWCNTs	SiGe/MWCNTs
R_o	2.28	1.96
R_c	3.30	2.64
R_s	26.31	12.94
R_{ct}	21.86	22.95
